# Whole genome sequencing of methicillin-resistant *Staphylococcus aureus* clinical isolates from Terengganu, Malaysia, indicates the predominance of the EMRSA-15 (ST22-SCC*mec* IV) clone

**DOI:** 10.1038/s41598-024-54182-x

**Published:** 2024-02-12

**Authors:** Ainal Mardziah Che Hamzah, Ching Hoong Chew, Esra’a Ibrahim Al-Trad, Suat Moi Puah, Kek Heng Chua, Nor Iza A. Rahman, Salwani Ismail, Toshinari Maeda, Prasit Palittapongarnpim, Chew Chieng Yeo

**Affiliations:** 1 Faculty of Health Sciences, Universiti Sultan Zainal Abidin, 21300 Kuala Nerus, Terengganu Malaysia; 2 Centre for Research in Infectious Diseases and Biotechnology (CeRIDB), Faculty of Medicine, Universiti Sultan Zainal Abidin, 20400 Kuala Terengganu, Terengganu Malaysia; 3 Department of Biomedical Science, Faculty of Medicine, Universiti Malaya, 50603 Kuala Lumpur, Malaysia; 4 Department of Biological Functions and Engineering, Graduate School of Life Science and Systems Engineering, Kyushu Institute of Technology, 2-4 Hibikino, Wakamatsu-Ku, Kitakyushu, 808-0196 Japan; 5 Pornchai Matangkasombut Center for Microbial Genomics (CENMIG), Department of Microbiology, Faculty of Science, Mahidol University, Bangkok, 10400 Thailand; 6 Faculty of Allied Medical Sciences, Jadara University, Irbid, Jordan

**Keywords:** Antimicrobial resistance, Bacterial genomics, Pathogens

## Abstract

Despite the importance of methicillin-resistant *Staphylococcus aureus* (MRSA) as a priority nosocomial pathogen, the genome sequences of Malaysian MRSA isolates are currently limited to a small pool of samples. Here, we present the genome sequence analyses of 88 clinical MRSA isolates obtained from the main tertiary hospital in Terengganu, Malaysia in 2016–2020, to obtain in-depth insights into their characteristics. The EMRSA-15 (ST22-SCC*mec* IV) clone of the clonal complex 22 (CC22) lineage was predominant with a total of 61 (69.3%) isolates. Earlier reports from other Malaysian hospitals indicated the predominance of the ST239 clone, but only two (2.3%) isolates were identified in this study. Two Indian-origin clones, the Bengal Bay clone ST772-SCC*mec* V (*n* = 2) and ST672 (*n* = 10) were also detected, with most of the ST672 isolates obtained in 2020 (*n* = 7). Two new STs were found, with one isolate each, and were designated ST7879 and ST7883. From the core genome phylogenetic tree, the HSNZ MRSA isolates could be grouped into seven clades. Antimicrobial phenotype-genotype concordance was high (> 95%), indicating the accuracy of WGS in predicting most resistances. Majority of the MRSA isolates were found to harbor more than 10 virulence genes, demonstrating their pathogenic nature.

## Introduction

As a major nosocomial pathogen, methicillin-resistant *Staphylococcus aureus* (MRSA) sparks considerable interest and has been the focus of multiple whole genome sequencing (WGS) studies. Many WGS studies on MRSA entail outbreak investigations^1–3^, indicating the high transmissibility of this microbe and the consequent danger of outbreak eruption. In addition, the highly virulent and multidrug-resistant nature of MRSA imposes a considerable burden on the medical community, emphasizing its importance as a bacterial pathogen. Infections caused by MRSA strains are usually associated with prolonged hospitalization and high treatment costs. A positive MRSA test can result in an additional 6.6 days of hospitalization and a USD 9,237 post-discharge expense^4^. A nationwide study in Japan involving data from inpatients at 1,133 acute care hospitals between April 1, 2014 and March 31, 2015, projected the overall incremental burden of MRSA to be around USD 2 billion, with a length of hospitalization of 4.34 million days^5^. Compared to methicillin-susceptible *S. aureus* (MSSA), patients with MRSA infections have higher morbidity and mortality rates. For example, a cohort study conducted in India found that MSSA patients fared better in terms of survival than MRSA patients^6^. Additionally, MRSA patients also have a much greater incidence of sequelae such as sepsis and multiorgan dysfunction ^6^. In Malaysia, the mortality rate of MRSA infection was reported at 11.76% (6/51) among patients admitted to the general surgical wards of Hospital Tuanku Jaafar (HTJ)^7^ and 37.3% (25/67) among patients with MRSA bacteremia at University Malaya Medical Centre (UMMC)^8^.

The National Surveillance of Antimicrobial Resistance (NSAR) program in Malaysia tracks and reports on nine pathogen species every year, including MRSA. The MRSA prevalence rates typically varied between 15 and 20% in years 2011–2020, but in 2021 they only reached 7%^9^. Although the incidence of MRSA in Malaysian hospitals appears to be on the decline, vigilant surveillance of this pathogen is still required. Even in non-outbreak circumstances, MRSA has a number of potential transmission events involving healthcare personnels, patients, and the environment that could ultimately result in an outbreak^10^. Thus, regular surveillance of MRSA is required to maintain control over this infection. Whole genome research involving MRSA strains is still limited in Malaysia and typically includes a small number of samples^11^. In this study, we used WGS to examine 88 clinical MRSA isolates collected over a period of five years (2016 – 2020) from a tertiary hospital in the state of Terengganu, Malaysia, to provide a comprehensive genomic insight of these isolates as well as to determine their population structure and strain relatedness. A further 18 MRSA genomes from Malaysia available in GenBank were also included in the analyses.

## Results

### Antimicrobial susceptibility of the HSNZ MRSA isolates (2016–2020)

A total of 197 MRSA isolates were collected from the Microbiology Laboratory of Hospital Sultanah Nur Zahirah (HSNZ), the main public tertiary hospital in the state of Terengganu, Malaysia from July 2016 to December 2020 (but with no collection in 2018). The antimicrobial resistance rates along with the prevalence of the macrolide-lincosamide-streptogrammin B (MLS_B_) resistance phenotypes and multidrug resistance (MDR) among these isolates are indicated in Table 1. The MRSA isolates exhibited high levels of phenotypic resistance to the following antimicrobial classes: beta-lactams (> 90% for cefoxitin, oxacillin, penicillin, and cefoperazone), fluoroquinolones (78.7% for ciprofloxacin, and 74.6% for moxifloxacin), and MLS_B_ (62.9% for erythromycin, and 58.9% for clindamycin). All isolates were susceptible to glycopeptides, linezolid, and ceftaroline, whereas resistance rates for other antibiotics ranged from 1 to 19% (Table 1). Inducible clindamycin resistance (iMLS_B_ or D-test positive) was the most prevalent MLS_B_ phenotype, occurring in 52.3% (103/197) of the isolates, whereas constitutive resistance (cMLS_B_ or resistance to both erythromycin and clindamycin) and the MS phenotype (D-test negative) were observed much less frequently at 6.6% (13/197) and 4.0% (8/197), respectively.

**Table 1:** Antimicrobial resistance characteristics of the HSNZ MRSA isolates obtained in 2016–2020.

Antimicrobial	No. of isolates per year				Total no. of isolates (*n* = 197) (%)
	2016 (*n* = 69) (%)	2017 (*n* = 69) (%)	2019 (*n* = 43) (%)	2020 (*n* = 16) (%)	
Beta-lactam					
Cefoxitin	69 (100)	69 (100)	43 (100)	16 (100)	197 (100)
Oxacillin	69 (100)	65 (94.2)	38 (88.4)	14 (87.5)	186 (94.4)
Penicillin	69 (100)	68 (98.6)	43 (100)	16 (100)	196 (99.5)
Cefoperazone	65 (94.2)	64 (92.8)	36 (83.7)	14 (87.5)	179 (90.9)
Fluoroquinolone					
Ciproloxacin	52 (75.4)	54 (78.3)	35 (81.4)	14 (87.5)	155 (78.7)
Moxifloxacin	52 (75.4)	54 (78.3)	28 (65.1)	13 (81.3)	147 (74.6)
Macrolide					
Erythromycin	48 (69.6)	51 (73.9)	17 (39.5)	8 (50.0)	124 (62.9)
Lincosamide					
Clindamycin	46 (66.7)	46 (66.7)	17 (39.5)	7 (43.8)	116 (58.9)
Aminoglycoside					
Gentamicin	15 (21.7)	11 (15.9)	5 (11.6)	6 (37.5)	37 (18.8)
Amikacin	11 (15.9)	4 (5.8)	0	0	15 (7.6)
Folate inhibitor					
Trimethoprim-sulfamethoxazole	8 (11.6)	2 (2.9)	1 (2.3)	0	11 (5.6)
Fucidane					
Fusidic acid	7 (10.1)	12 (17.4)	3 (7.0)	1 (6.3)	23 (11.7)
Tetracycline					
Tetracycline	9 (13.0)	4 (5.8)	2 (4.7)	0	15 (7.6)
Doxycycline	6 (8.7)	1 (1.4)	1 (2.3)	0	8 (4.1)
Minocycline	3 (4.3)	0	0	0	3 (1.5)
Glycylcycline					
Tigecycline	1 (1.4)	4 (5.8)	0	5 (31.3)	10 (5.1)
Phenicol					
Chloramphenicol	1 (1.4)	6 (8.7)	1 (2.3)	1 (6.3)	9 (4.6)
Monoxycarbolic acid					
Mupirocin	1 (1.4)	1 (1.4)	0	0	2 (1.0)
Ansamycin					
Rifampin	2 (2.9)	0	1 (2.3)	0	3 (1.5)
Glycopeptide					
Vancomycin	0	0	0	0	0
Teicoplanin	0	0	0	0	0
Oxazolidinone					
Linezolid	0	0	0	0	0
Streptogramin					
Quinupristin-dalfopristin	0	0	0	0	0
Anti-MRSA cephalosporin					
Ceftaroline	0	0	0	0	0
MLS_B_ resistance phenotype					
cMLS_B_	7 (10.1)	5 (7.2)	1 (2.3)	0	13 (6.6)
iMLSB	39 (56.5)	41 (59.4)	16 (37.2)	7 (43.8)	103 (52.3)
MS	2 (2.9)	5 (7.2)	0	1 (6.3)	8 (4.1)
Susceptible	21 (30.4)	18 (26.1)	26 (60.5)	8 (50.0)	73 (37.1)
No. of MDR isolates	54 (78.3)	57 (82.6)	21 (48.8)	14 (87.5)	146 (74.1)
No. of non-MDR isolates	15 (21.7)	12 (17.4)	22 (51.2)	2 (12.5)	51 (25.9)
No. of MDR isolates selected for whole genome sequencing in this study	25	29	20	14	88

The majority of the MRSA isolates (146/197; 74.1%) were multidrug-resistant (MDR), exhibiting resistance primarily against four antibiotic classes. One MDR isolate, SauR65, had a notable level of resistance with up to 10 antibiotic classes. The rest of the isolates (25.9%, 51/197) showed resistance only to beta-lactams (*n* = 15) or beta-lactams in combination with another antibiotic class (*n* = 36) (Table 1). It should be noted that the isolates obtained in 2020 were considerably fewer in quantity (*n* = 16) due to the Covid-19 pandemic which restricted our access to the hospital laboratory.

### Genome assembly quality

Out of the 146 MDR MRSA isolates that were collected, a total of 88 MDR MRSA isolates were randomly chosen to be sequenced on the short-read Illumina or DNBSeq platforms with the mean sequencing coverage of 205 × . The average number of contigs among the sequenced isolates was 53 (Min = 24; Max = 173). The contigs had an average total length of 2,819,047 bp, while the GC content averaged at 32.7%. Additionally, the average N50 value was 189,136, while the average L50 value was six. The details of the assembly qualities and statistics are available in Supplementary Table S2.

### Epidemiological characteristics of the sequenced MRSA isolates

**Figure 1:**
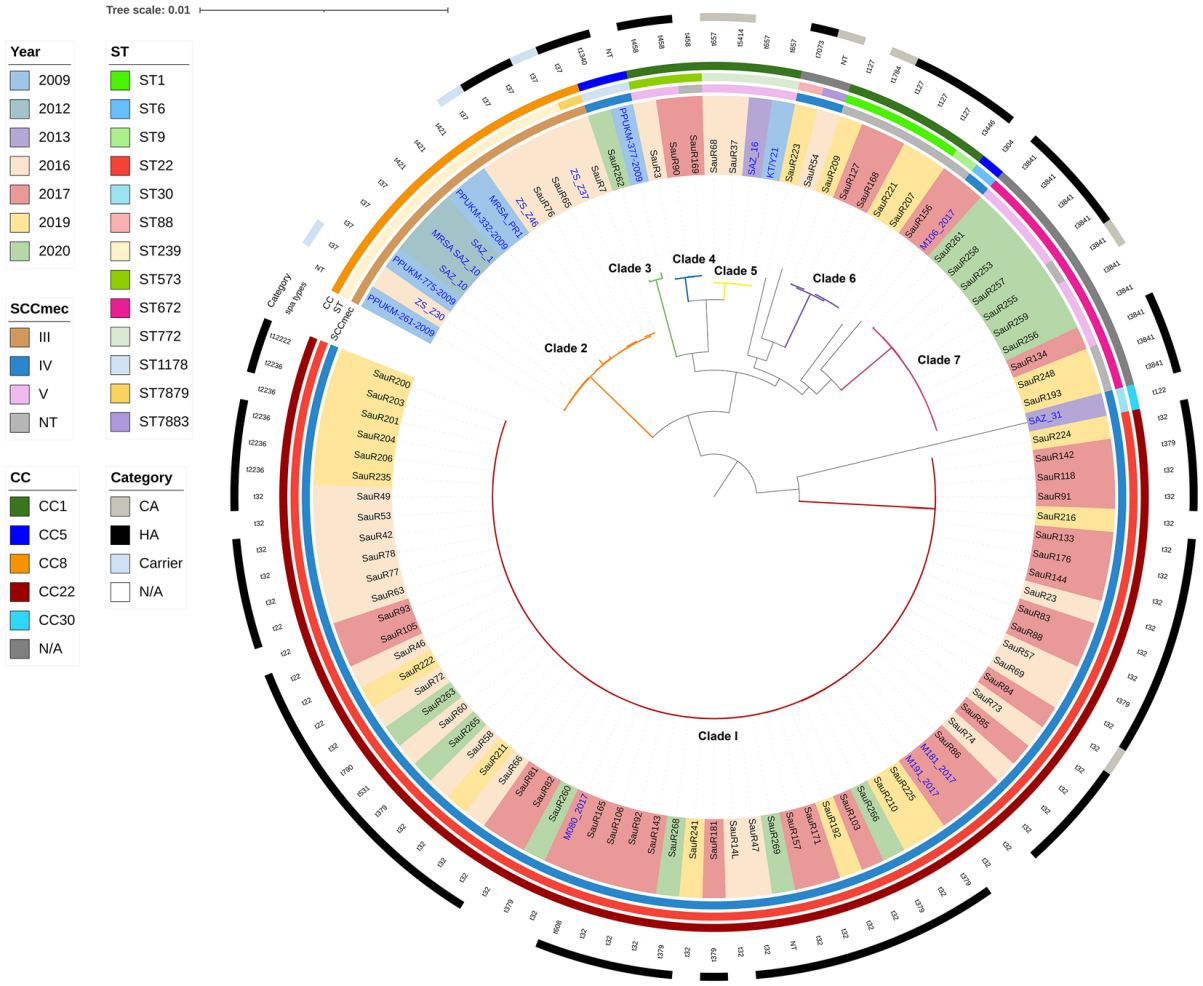
Core genome maximum-likelihood phylogenetic tree of the 88 MRSA isolates obtained from HSNZ (from 2016 to 2020) along with 18 other Malaysian MRSA isolates that were available in GenBank (labelled in blue fonts; see Supplementary Table S1 for details of these isolates). Seven clades could be identified from the phylogenetic tree and these are represented by the following color-coded branches: Clade I (in red), Clade II (orange), Clade III (green), Clade IV (blue), Clade V (yellow), Clade VI (purple), and Clade VII (magenta). The color blocks with the names of the isolates indicate the year of isolation as in the top left legend. Other information included in the diagram are (from inner to outer circle): SCC*mec* type, sequence type (ST), clonal complex (CC), *spa* type, and category (i.e., whether they are hospital-acquired (HA), community-acquired (CA), or carrier). N/A = not available.

Molecular typing using whole genome data identified three main SCC*mec* types, 11 sequence types (STs) and 19 *spa* types (Fig. 1). ST22-SCC*mec* IV was identified as the predominant clone in 61 isolates (69.3%), followed by ST672-SCC*mec* V in seven isolates (7.9%). The in silico SCC*mec* typing was unable to type 10 isolates belonging to ST1 (*n* = 5), ST672 (*n* = 3), ST9 (*n* = 1), and ST573 (*n* = 1). SauR7 and SauR54 isolates showed new allelic profiles, which ST7879 and ST7883 were respectively assigned to them by PubMLST. These STs could be grouped into four different clonal complexes (CCs), with ST22 clustered into CC22 which constituted the largest group (*n* = 61). ST1, ST9, ST573 and ST772 belonged to CC1, ST1178 belonged to CC5, and ST239 along with the new ST7879 belonged to CC8. ST7879 only differed by a single locus to ST239, i.e., the *pta* allele. However, ST88, ST672 and the new ST7883 do not belong to any known CCs. Of the 19 identified *spa* types, t032 was the most common with 38 (43.2%) isolates, all of which were found exclusively in ST22. This was followed by t3841 (*n* = 10, 11.4%) and t379 (*n* = 8, 9.1%), which were found in ST672 and ST22 isolates, respectively. Two isolates were *spa* untypeable, one of which was the novel ST7883 isolate.

Based on the clinical records, the 88 MRSA isolates were classified either as hospital-associated (HA-MRSA) or community-associated MRSA (CA-MRSA) following the criteria defined by the Centers for Disease Control and Prevention (CDC)^12^. HA-MRSA isolates made up the majority with 70 isolates, while CA-MRSA accounted for only six isolates, whereas 12 isolates could not be categorized (as these isolates originated from samples provided to the HSNZ laboratory from district clinics and hospitals and were thus without detailed clinical data). Two of the six CA-MRSA isolates belonged to ST772, and the remaining four CA-MRSA isolates belonged to ST1, ST22, ST672, and ST7883 each. The isolates harboring SCC*mec* III and those belonging to ST9, ST88, ST573, and ST1178 were all classified as HA-MRSA.

### Pan genome and phylogenetic analysis

The pan genome analysis of the 88 MRSA isolates from HSNZ revealed a total of 4,742 genes, which consisted of 1,973 core genes, 157 soft core genes, 783 shell genes and 1,829 cloud genes. The 88 MRSA isolates from the present study along with the 18 Malaysian database isolates could be grouped into seven clades based on their STs (Fig. 1). Clade I was the largest and was represented by the 61 ST22 isolates from HSNZ, and three other ST22 Malaysian isolates obtained from GenBank. Most of these HSNZ isolates were HA-MRSA strains. In Clade II, the novel ST7879 isolate was clustered with 12 ST239 isolates, demonstrating a close relationship between the two STs, which belonged to CC8. All these CC8 isolates also harbored SCC*mec* type III. Ten of the remaining 18 Malaysian MRSA database isolates were clustered in Clade II (ST239), one in Clade III (ST1178), two in Clade V (ST772), while two did not cluster in any clade. Clades IV (ST573), VI (ST1), and VII (ST672) were fully represented by our HSNZ isolates. Although ST1, ST9, ST573, and ST772 belonged to CC1, they appeared to be separate lineages and were not closely related. Notably, while the ST239-SCC*mec* III clone dominated the Malaysian MRSA isolates collected in 2009 and 2012, only two of our isolates belonged to this clone, both of which were obtained in 2016.

Among the isolates collected in 2020, there was a sudden increase in the number of ST672 isolates, with half of them (*n* = 7/14) belonging to this clone (Fig. 1). In contrast, only one ST672 isolate was found out of the 29 isolates that were sequenced in 2017, and two out of 20 in 2019. Even though only 14 MRSA isolates from 2020 were sequenced due to the small pool of isolates that could be obtained that year, this sudden increase in the proportion of ST672 isolates that were obtained in 2020 is certainly noteworthy. Seven of these ST672 isolates harbored SCC*mec* type V whereas the remaining three were non-typeable (Fig. 1).

### Distribution of antimicrobial resistance genes

**Figure 2:**
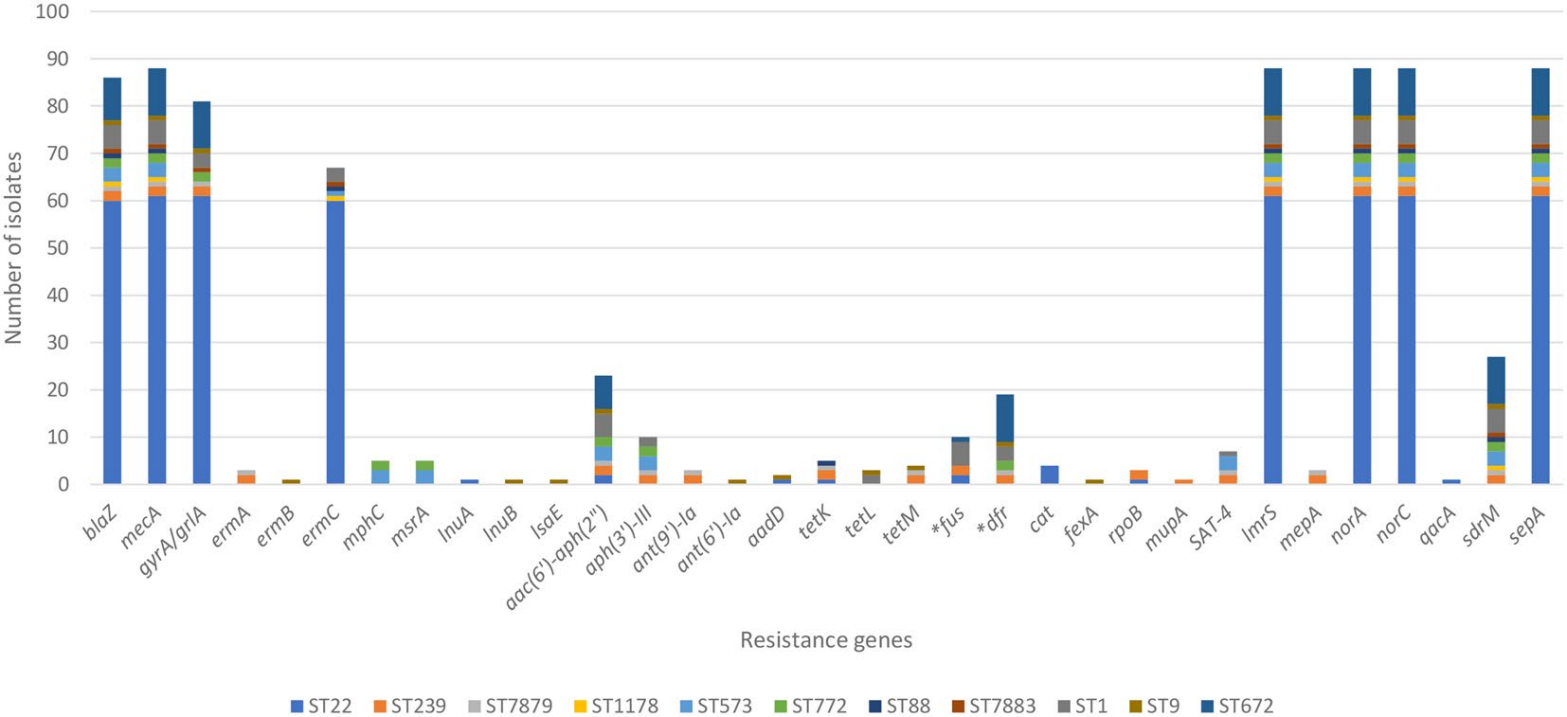
Distribution of antimicrobial resistance determinants amongst the 88 MRSA isolates from HSNZ according to their STs. Genes marked with asterisks represent genes with variations in the form of mutations that were predicted to cause resistance and are grouped together.

**Figure 3:**
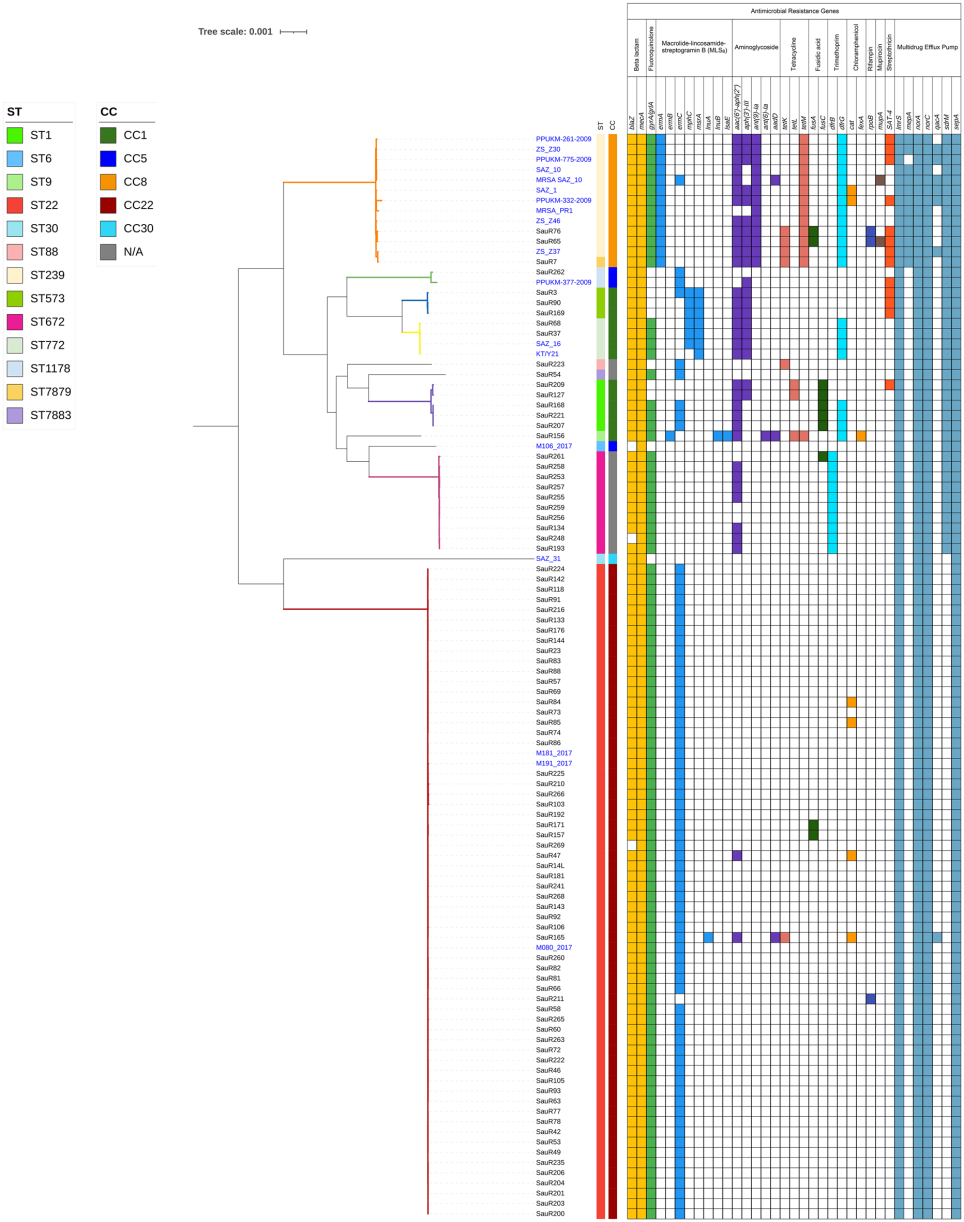
Antimicrobial resistance determinants amongst the 88 MRSA isolates sequenced in this study along with another 18 Malaysian MRSA isolates obtained from GenBank (labelled in blue fonts). The core genome maximum-likelihood phylogenetic tree of the isolates is shown on the left and the branches are colored according to the seven clades identified in Fig. 1. The presence of a particular resistance determinant is indicated by a colored box. Genes marked with asterisks (*) represent host genes in which mutations were detected that would lead to resistance.

The distribution of antibiotic resistance genes is shown in Fig. 2. A total of 35 genes causing resistance to different antibiotic agents including beta lactams (*blaZ* and *mecA*), MLS_B_ antibiotics (*ermABC*, *msrA*, *mphC*, *lnuAB*, and *lsaE*), aminoglycosides [*aac(6’)-aph(2″)*, *aph(3’)-III*, *ant(9)-Ia*, *ant(6)-Ia*, and *aadD*], streptothricin (*SAT-4*), tetracyclines (*tetK, tetL*, and *tetM*), fusidic acid (*fusC*), trimethoprim (*dfrG*), chloramphenicol (*cat* and *fexA*) and mupirocin (*mupA*) were identified (Fig. 3). Chromosomal point mutations including *gyrA/grlA* (fluoroquinolone), *fusA* (fusidic acid), *dfrB* (trimethoprim), *rpoB* (rifampin) and genes encoding multidrug efflux pumps such as *lmrS*, *mepA*, *norA*, *norC*, *qacA*, *sdrM*, and *sepA* were also identified.

In general, ST22 isolates appeared to be more susceptible. *ermC* was detected in the ST22 isolates at a high frequency (*n* = 60). This gene is located on a small RepL-type plasmid (2.4–2.7 kb) in the ST22 isolates^13^ as well as other isolates. The *cat* gene that mediates resistance to chloramphenicol was identified only in four isolates of ST22 (Fig. 3). SauR165 (ST22 *cat*-positive isolate) carried more resistance genes (*n* = 14) compared to other ST22 isolates (*n* = 7–10). SauR165 was found to harbor a 28.6 kb RepA_N-type plasmid, pSauR165-1, which encode six antibiotic resistance genes including *cat*, *tetK*, *ant(4’’)-Ib*, *aac(6’)-Ie-aph(2’’)-Ia*, *lnuA* and *qacA*, along with the *cadDX* cadmium resistance genes^13^.

ST88, ST1178, and ST7883 isolates had almost similar resistomes as the ST22 isolates but the ST88 isolate, SauR223, had an additional *tetK* gene. Among our isolates, those belonging to ST239 and ST7879 were predicted to be highly resistant compared to other isolates. They carried *ermA*, *mepA*, and unique combinations of aminoglycoside and tetracycline resistance genes that were not found in other STs. One of the ST239 isolates, SauR65, was the only isolate harboring the *mupA* gene in our collection. The other *mupA*-positive isolate, MRSA SAZ_10 (accession no. SWED00000000), was also ST239 and from HSNZ in 2012 but was sequenced in an earlier study.

ST573 (*n* = 3) and ST772 (*n* = 2) isolates carried more than 10 resistance genes including *msrA* and *mphC* macrolide resistance genes and aminoglycoside resistance genes as a combination of *aac(6’)-aph(2″)* and *aph(3’)-III*. All ST573 and ST772 isolates harbored the *SAT-4* and *dfrG* resistance genes, respectively. *dfrG* was also found in two other ST772 isolates that were obtained from HSNZ previously (KT/Y21 in 2009 and SAZ_16 in 2013). We recently reported the complete genome sequence of one of the ST573 isolates, SauR3 (CP098727), by hybrid assembly of Illumina and Oxford Nanopore sequence reads^14^. SauR3 carried a 42.9 kb plasmid (pSauR3-1) with an approximately 14 kb genomic island containing resistance genes including the *bla* operon in a full-length Tn*552*, *mphC*, *msrA*, *SAT-4*, and *aadE-aph(3’’)-IIIa*. Besides, SauR3 also harbored the small, 2.5 kb RepL-type plasmid encoding *ermC* (designated pSauR3-3) which was responsible for its inducible iMLS_B_ phenotype. Another interesting feature of the SauR3 genome is the presence of a SCC*mec* type V (5C2&5) variant which also encodes the *aac(6’)-aph(2’’)* aminoglycoside-resistance genes flanked by IS*431*^14^. However, tracing the presence of such genetic features in the other isolates of ST573 (SauR90 and SauR169) was not possible using short-read sequences alone. Nevertheless, both SauR90 and SauR169 did not harbor the *ermC*-encoding RepL-type plasmid and displayed the MS phenotype^13^.

The *fusC* gene was primarily associated with ST1, with five out of six *fusC*-positive isolates belonging to this ST. The other isolate harboring *fusC*, SauR261, was ST672 (Fig. 3). Three of the ST1 isolates (SauR168, SauR207, and SauR221) had a combination of the *ermC* and *dfrG* genes. The *aac(6’)-aph(2’’)* genes were identified in all ST1 isolates and in two of these isolates (SauR127 and SauR209), an additional *aph(3’)-III* gene in combination with *tetL* were found. SauR209 also had an additional *SAT-4* streptothricin resistance gene which was not found in the other ST1 isolates.

The findings revealed that SauR156 (the only ST9 isolate) carried 18 resistance genes, including some genes that were not found in other STs such as *ermB*, *lnuB*, *lsaE*, and *fexA*. Furthermore, an 11.3 kb PriCT_1 plasmid (pSauR156-1), which encodes *ermB*, *tetL*, and *aadD* was detected in SauR156^13^.

Similar to ST22 isolates, the ST672 isolates also appeared to be more susceptible to antimicrobials, with resistance genes detected in the range of nine to ten genes. Nine of the ten ST672 isolates harbored the *blaZ* gene in the 6,545 bp transposon Tn*552* which was encoded on a 20.7 kb plasmid that was highly similar to pMW2, a 20,654 bp Rep_3-type plasmid initially described in MRSA strain MW2 isolated in USA in 1998^13,15^. The sole *blaZ*-negative ST672 isolate, SauR248, was plasmid-free. The *dfrB* gene was identified strictly among these isolates, with all 10 of them carrying this gene. Additionally, seven isolates possessed the *aac(6’)-aph(2″)* aminoglycoside resistance gene. In contrast to the ST22 isolates, the ST672 isolates do not harbor the *ermC* MLS_B_ resistance gene.

### Correspondence of phenotypic and genotypic resistance

Phenotypic resistance of the 88 MRSA isolates from HSNZ was compared with genotype prediction from WGS data for 10 antibiotic classes. Generally, the concordance rate was determined at > 95% for all antibiotics tested in this study (Table 2). In isolates demonstrating phenotypic resistance to trimethoprim-sulfamethoxazole (SXT) that were sequenced (*n* = 9), WGS analysis using ResFinder and CARD databases identified the *dfrG* gene as the resistance determinant for trimethoprim but the acquired sulfonamide resistance genes *sul1*, *sul2* and *sul3* were absent. To elucidate the resistance mechanism associated with sulfamethoxazole, we conducted BLAST searches and found mutations in the *folP* gene, which encodes the dihydropteroate synthase (DHPS) enzyme. The resistant ST239 (*n* = 2) and ST7879 (*n* = 1) isolates exhibited five mutations (F17L, T28S, T59S, L64M, and KE257_Dup), while the remaining resistant isolates (*n* = 6) had three mutations (T28S, T59S, and L64M). Previous studies have shown that these mutations confer resistance to sulfamethoxazole, thereby explaining the observed SXT resistance phenotype^16,17^.

**Table 2:** Concordance rate between antimicrobial phenotypes and predicted genotypes.

Antibiotic class	Antibiotic	Resistant isolates	False positive	False negative	Concordance (%)	Resistance determinants
Beta-lactam	Penicillin	88	0	2	97.7	*blaZ*
	Cefoxitin	88	0	0	100	*mecA*
MLS_B_	Erythromycin/ clindamycin/ streptogramin	76	0	1	98.9	*erm, msr*
Fluoroquinolone	Ciprofloxacin/ moxifloxacin	82	0	0	100	Mutations in *gyrA/grlA*, *norA*
Aminoglycoside	Amikacin/ gentamicin	26	0	2	97.7	*aac(6’)-aph(2″), aph(3’)-III, ant(9)-Ia, ant(6)-Ia, aadD*
Tetracycline	Tetracycline/ doxycycline/ minocycline	9	0	1	98.9	*tet*
Folate inhibitor	Trimethoprim-sulfamethoxazole	8	1	0	98.9	*dfrG*
Phenicol	Chloramphenicol	5	0	0	100	*cat*
Fucidane	Fusidic acid	13	0	3	96.6	*fus*
Ansamycin	Rifampicin	3	0	0	100	Mutation in *rpoB*
Monoxycarbolic acid	Mupirocin	1	0	0	100	*mupA*

### Distribution of virulence genes

VirulenceFinder identified a total of 26 distinct virulence genes among the 88 MRSA isolates, which consisted of host immunity genes (*sak* and *scn*), exoenzyme genes (*aur* and *splABE*), and toxin genes such as epidermal cell differentiation (*edinA*), gamma hemolysins (*hlgABC*), leukotoxin (*lukED*), Panton-Valentine leukocidin (*lukFS*) and enterotoxins (*sea*—*sec*, *seg*—*sei*, *sek*—*seq*, and *seu*). The majority of the isolates (96.6%, 85/88) carried more than 10 virulence genes in their genomes.

**Figure 4:**
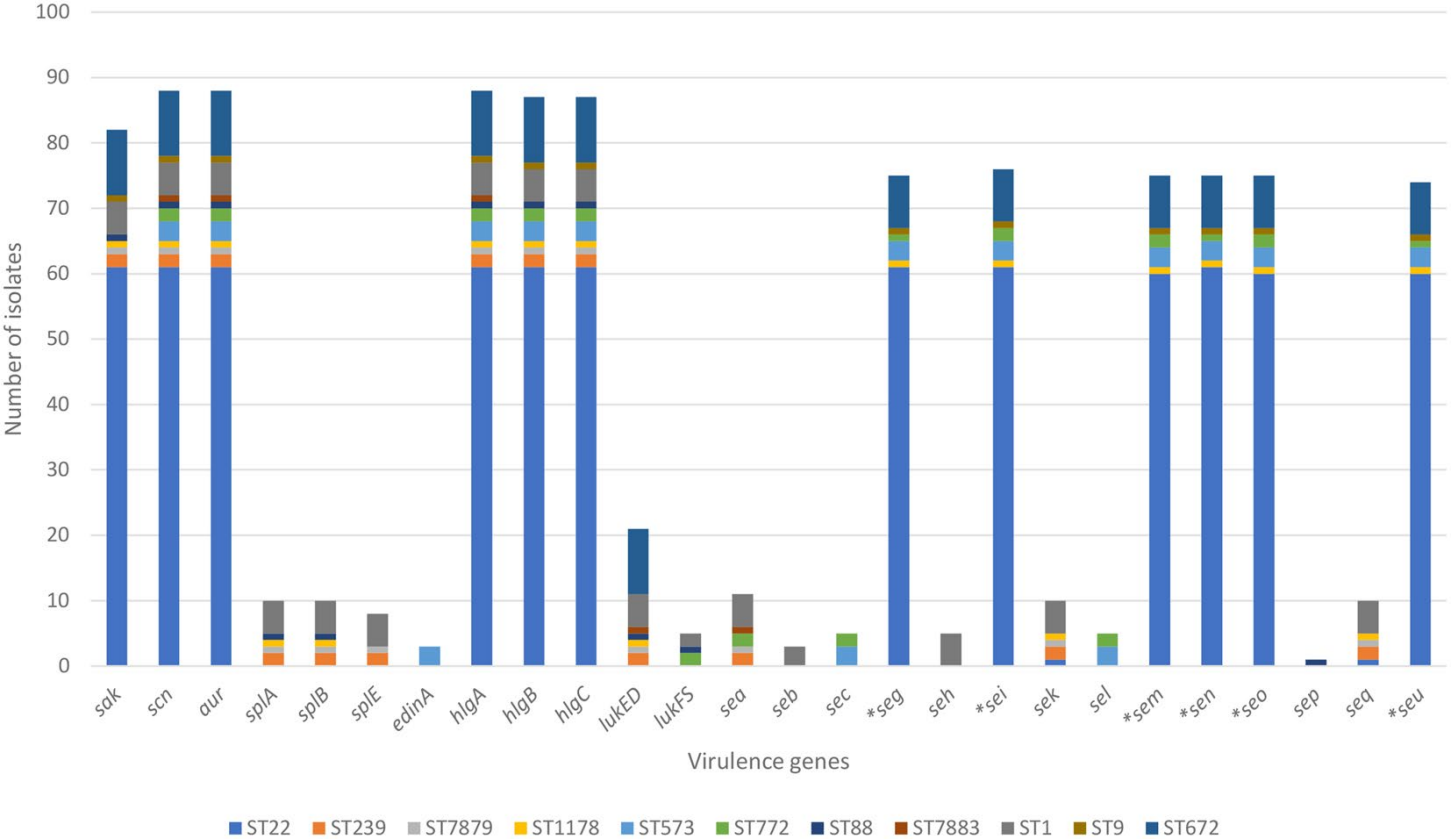
Distribution of virulence genes amongst the 88 MRSA isolates from HSNZ that were sequenced in this study according to their STs. Genes marked with asterisks (*) represent the genes from the enterotoxin gene cluster (egc).

**Figure 5:**
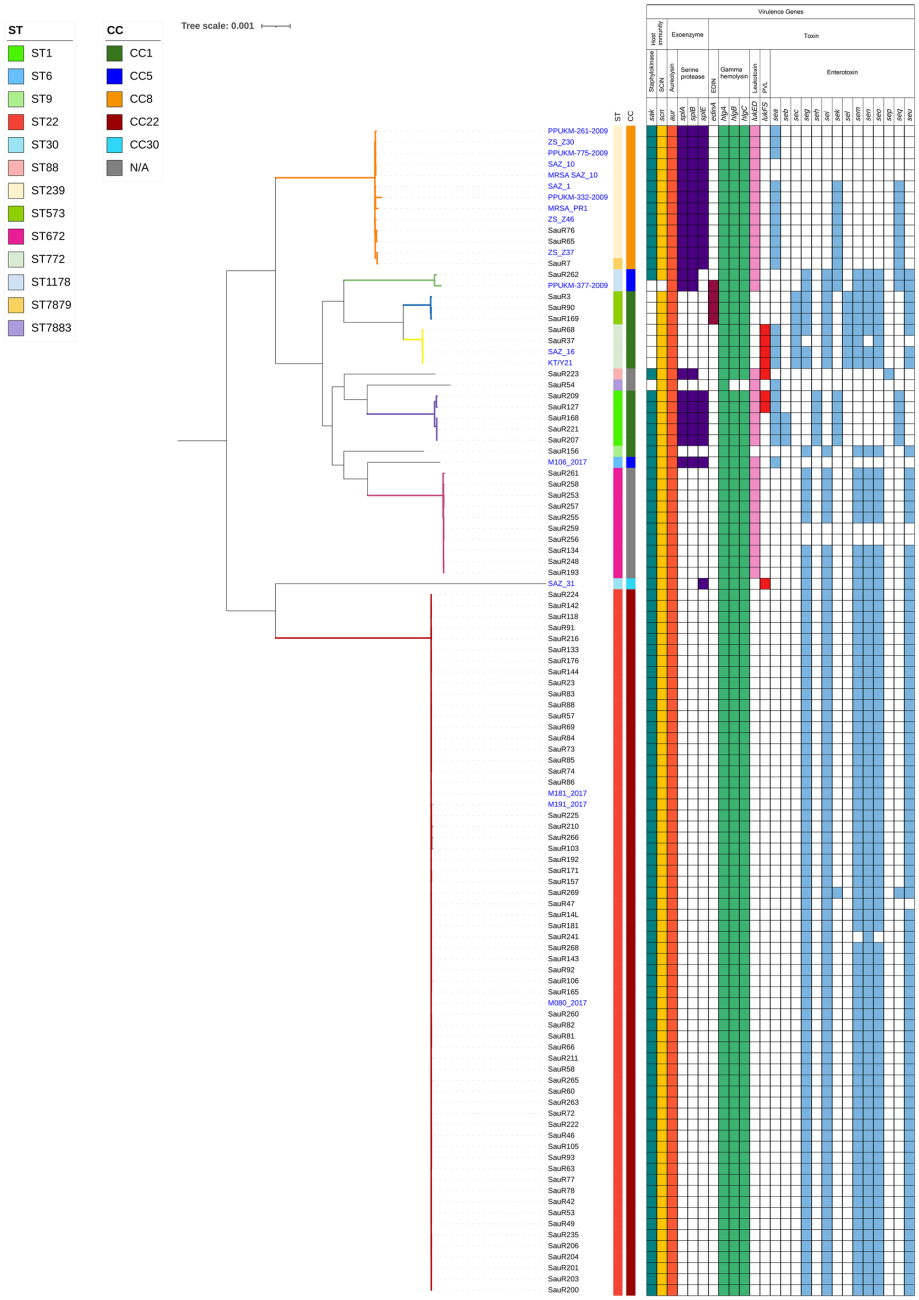
Virulence genes among the 88 MRSA isolates from HSNZ (2016–2020) along with another 18 Malaysian MRSA isolates obtained from GenBank (labelled in blue fonts). The core genome maximum-likelihood phylogenetic tree of the isolates is shown on the left with the branches colored according to the seven clades identified in Fig. 1. The presence of a particular virulence gene is marked by a colored box. SCIN = staphylococcal complement inhibitor; EDIN = epidermal cell differentiation inhibitor; PVL = Panton-Valentine leucocidin.

Genes such as *sak*, *scn*, *aur*, *hlgABC*, and genes of the enterotoxin gene cluster (egc) including *seg*, *sei*, *sem*, *sen*, *seo,* and *seu*, were widely present among the HSNZ as well as the other Malaysian isolates (Figs. 4 and 5, respectively). These genes constituted the virulence profiles of most (*n* = 58) ST22 isolates and the single ST9 isolate. Of the 61 ST22 isolates, only three differed slightly in their enterotoxin gene profile. One isolate (SauR269) had additional *sek* and *seq* genes, one (SauR47) lacked the *seu* gene, and another (SauR241) lacked the *sem* and *seo* genes. The virulence profiles of ST239 (SauR65 and SauR76) and ST7879 (SauR7) isolates were comparable, with the exoenzyme *splABE* and leukotoxin *lukED* genes present in all three isolates. These three isolates also contained the combination of *sea*, *sek* and *seq* genes in place of the missing egc genes. In contrast, the *sek* and *seq* genes were less common among the Malaysian ST239 isolates in the database, with five of them (i.e., PPUKM-261-2009, PPUKM-775-2009, ZS_Z30, SAZ_10 and MRSA SAZ_10) lacking these genes. Furthermore, the *sea* gene was also absent in both SAZ_10 and MRSA SAZ_10.

The *lukED*, *splAB*, *sek*, and *seq* genes were also present in the genome of the ST1178 isolate, SauR262. However, unlike ST239 and ST7879, this isolate contained the egc genes. Notably, SauR262 carried the highest number of virulence genes in this study, with a total of 17 genes identified in its genome. The only other Malaysian ST1178 genome which was obtained from GenBank, PPUKM-377-2009, was different from SauR262 in that it contained the *edinA* epidermal cell differentiation gene but lacked the *sak* and *scn* genes.

Both ST573 and ST772 isolates lacked the *sak* gene, which was found in most (*n* = 82) of the other MRSA isolates. In addition to the egc genes, these isolates also contained the *sec* and *sel* genes, which were not detected in any of the other isolates. Among the HSNZ isolates, the *edinA* gene was identified exclusively in ST573, whereas ST772 isolates also had the *lukFS* Panton-Valentine leukocidin and *sea* genes. The presence of enterotoxin genes was rare in the HSNZ ST88 isolate, which only possessed the single *sep* gene discovered in this study. This isolate was also one of the few to contain both *lukED* and *lukFS* genes.

Similar to ST88, the ST7883 isolate (SauR54) also lacked enterotoxin genes. SauR54 carried the least number of virulence genes, including *scn*, *aur*, *hlgA*, *lukED*, and *sea*. ST1 isolates displayed a diverse virulome, with up to 15 types of virulence genes identified across all five isolates. Their enterotoxin gene profiles were mostly similar to ST239 and ST7879, but with an additional *seh* gene. The *seh* gene was a unique feature of ST1 isolates, as it was not identified in other STs.

The *splABE* and *lukED* genes were also found in all five ST1 isolates. Two isolates (i.e., SauR127 and SauR209 also contained *lukFS*, while the remaining three *lukFS*-negative isolates (i.e., SauR168, SauR207, and SauR221) had the *seb* gene instead.

The virulome of ST672 isolates was comparable to that of ST22 and ST9 isolates with the addition of *lukED*. Except SauR256 and SauR259 isolates, which carried seven virulence genes and lacked the egc genes, most (*n* = 8) ST672 isolates carried 13 virulence genes (Fig. 5).

## Discussion

WGS has emerged as a successful approach for identifying the transmission and outbreaks of bacterial pathogens such as MRSA. Due to its multiple resistances and high pathogenicity, MRSA has remained a persistent threat since the 1960s, having significant detrimental effect on public health. Therefore, continuous surveillance of this pathogen is essential to contain its spread. In this study, 88 clinical MDR MRSA isolates were analyzed using WGS to obtain information about the population and clonal composition of circulating MRSA isolates in HSNZ, the main tertiary hospital in Terengganu in 2016–2020.

Molecular epidemiology of the 88 MRSA isolates in this study revealed that the ST22-SCC*mec* IV, also known as epidemic MRSA-15 (EMRSA-15) was the most prevalent clone, with 61 (69.3%) isolates. EMRSA-15 is a major clone that has exhibited considerable success on a global scale and was previously highly prevalent in the UK and Ireland^18^. Studies in Singapore showed that in the 1980s and 1990s, the ST239-SCC*mec* III clone was predominant, but starting from 2003 when the first ST22-SCC*mec* IV clone was introduced, the ST239-SCC*mec* III clone began to be slowly displaced by ST22-SCC*mec* IV which by the mid-2010’s had established itself in most Singapore hospitals^19,20^. In Malaysia, a similar clonal displacement event had also likely occurred. During the mid-2000’s, the ST239-SCC*mec* III clone was predominant in Malaysia^21,22^ but from early 2010’s onwards, there were reports of the growing proportion of ST22-SCC*mec* IV in Malaysian hospitals^23^. Interestingly, most of the Malaysian ST239-SCC*mec* III genomes that were obtained from GenBank (*n* = 10) originated in 2009 and 2012, perhaps reflecting their predominance during that time. By the mid 2010’s, the ST22-SCC*mec* IV clone appeared to predominate with a study from the University of Malaya Medical Centre (UMMC) in Kuala Lumpur, Malaysia, indicating that 55.6% of the 99 MRSA isolates collected between 2014–2015 belonging to this particular clone^24^. The predominance of the ST22-SCC*mec* IV clone was also clearly demonstrated in the MRSA isolates from HSNZ, Terengganu in 2016–2020. Despite the ST22-SCC*mec* IV clone being more susceptible to antibiotics compared to ST239-SCC*mec* III^19,23^, the success of the ST22 clone could be due to its physiological characteristics. In vitro studies indicate that ST22 is a fit clone relative to ST239, with a higher growth rate and resistance to desiccation^25^. Besides, the smaller size of SCC*mec* IV as compared to SCC*mec* III could enable it to be more efficiently transmitted via horizontal gene transfer^26,27^.

Among the MRSA isolates in this study, the presence of two Indian-origin clones, ST672-SCC*mec* V and the Bengal Bay clone ST772-SCC*mec* V, is noteworthy. Since the majority of the Bengal Bay clone in Norway was isolated from patients with ties to the Indian subcontinent^28^, it is possible that these two clones made its way to Malaysia through travelers from the same region. Although the ST772 Bengal Bay clone has previously been observed in Malaysian isolates^29,30^ including from HSNZ^31^, to the best of our knowledge, the detection of the ST672 clone has not been documented. However, it has been reported in neighboring Indonesia^32^. Interestingly, the ST672 clone was identified in just one isolate in 2017, two isolates in 2019, and seven in 2020, with majority of these isolates (7/10) harboring SCC*mec* V. This increasing prevalence of the ST672 clone suggests a potential upward trend. Continued surveillance is needed to confirm this and assess if another clonal displacement, like the ST239-SCC*mec* IV in the early 2010s, is occurring. The possible reason for this apparent prevalence of ST672 in HSNZ is currently unknown.

The detection of resistance determinants uncovered a total of 35 resistance genes associated with resistances to antiseptics and 12 antibiotics classes. Methicillin resistance among the MRSA isolates in this study is mediated by *mecA*, while the *mecA* homologue, *mecC*, was not detected. The *mecC* gene has so far been found primarily in European isolates of varying clonal lineages such as CC130, CC49, CC599, and CC1943^33^. To date, *mecC* has not been detected among human MRSA isolates in Malaysia. However, one recent study reported the detection of 15 *mecC*-positive livestock-associated MRSA (LA-MRSA) isolates in milk and nasal swab samples from dairy cattle farms^34^. Despite being a successful pandemic clone, ST22-SCC*mec* IV isolates in this study were among the most susceptible, exhibiting resistance to only beta lactams, fluoroquinolones, and MLS_B_ antibiotics, which is consistent with previous studies^19,23,35^. Conversely, ST239-SCC*mec* III and ST7879-SCC*mec* III isolates, which shared similar resistomes, were predicted that they would be the most resistant (resistance against aminoglycosides, tetracyclines, and nucleoside antibiotics). The Vienna/Hungarian/Brazilian clone ST239-SCC*mec* III, which has been around since the 1970s, is the oldest pandemic strain and is known to be multidrug-resistant^36,37^. The larger size of SCC*mec* III in comparison to SCC*mec* IV, enables it to carry a plethora of resistance genes^38^, thus explaining the observed multidrug resistance.

The solitary ST9 isolate in this study, SauR156, is particularly intriguing as ST9 strains are commonly LA-MRSA. Our finding that the ST9 isolate carried a high number of resistance genes corroborated previous reports that strains of this ST are exceptionally resistant^39,40^.

We found 95% concordance between phenotypic resistance and genotypic prediction, indicating the high accuracy of WGS in detecting antimicrobial resistance, as reported by other studies^41,42^. In addition to resistance genes, the MRSA isolates were also found to harbor numerous virulence genes. Across the 88 MRSA genomes, as many as 26 distinct genes were discovered, with *sak*, *scn*, *aur*, egc, and *hlg* genes being among the most frequently encountered. The *sak* gene encodes staphylokinase, a plasminogen activator with fibrinolytic function^43^, while the *scn* gene encodes staphylococcal complement inhibitor (SCIN), an immune modulating protein implicated in the prevention of phagocytosis^44^. Both *sak* and *scn* genes are located on the ϕSa3 prophage, and are components of the immune evasion cluster (IEC), which has been demonstrated to play an essential role in the colonization of human hosts^40,45^. The IEC is usually absent in animal isolates; hence the presence of these genes typically indicates human adaptation. Notably, the absence of the *scn* gene is considered a hallmark for LA-MRSA strains^46^. However, we found that this gene was present in our ST9 isolate, which is commonly associated with LA-MRSA. This indicates that the isolate has successfully adapted to and colonize the human host, eventually leading to clinical infection^47^. The high prevalence of egc genes observed in this study is consistent with findings from earlier studies^48,49^. Similarly, the widespread nature of gamma hemolysin across our isolates is also explained by the fact that it is a core genome-encoded toxin found in virtually all strains of *S. aureus*^50^. On the contrary, the occurrence of the leukotoxin genes, *lukED* encoding leukocidin ED and *lukFS* encoding Panton-Valentine leukocidin (PVL), was observed to be relatively rare. A previous review has indicated that the *lukED* genes are widely prevalent among *S. aureus*, particularly in epidemic strains^51^. However, in the present study, only a total of 21 isolates (23.9%) harbored the *lukED* genes, which were distributed among various sequence types (ST1: *n* = 5, ST88: *n* = 1, ST239: *n* = 2, ST672: *n* = 10, ST1178: *n* = 1, ST7879: *n* = 1, ST7883: *n* = 1). In addition, the *lukED* genes were not found in the ST22 isolates, which was consistent with a previous study^52^. Despite recent findings which described the emergence of hypervirulent ST22 strains carrying both PVL and *tst* genes^53,54^, the HSNZ ST22 isolates notably lacked these virulence factors. Interestingly, PVL- and TSST-1-positive ST22 strains were associated with SCC*mec* IVa^53,54^, whereas the HSNZ isolates belonged to the SCC*mec* IVh subtype, suggesting a genetic makeup distinctive from the hypervirulent strains despite their shared ST22 lineage. The PVL-encoded gene *lukFS* were identified only in the genomes of ST88 (SauR233), two ST772 (SauR37 and SauR68), and two ST1 (SauR127 and SauR209) isolates. Three Malaysian isolates from earlier studies were found to be PVL-positive, and these were SAZ_31 belonged to ST30, and SAZ_16 and KT/Y21 belonged to ST772. Earlier research had documented the carriage of PVL in ST88 strains^55^, while the ST772 Bengal Bay clone is known to be a PVL-positive clone^28^. Because PVL has been linked with severe disease^56,57^, carriage of this gene might indicate the high pathogenicity of ST88 and ST772 isolates.

## Conclusions

WGS of 88 MDR MRSA isolates obtained from 2016 to 2020 from HSNZ in Terengganu has given us important insights into their genome characteristics, carriage of antibiotic resistance and virulence genes, and their clonal lineages. The ST22-SCC*mec* IV (EMRSA-15) clone was predominant in HSNZ (*n* = 61/88; 69.3%), reflecting other studies done in Malaysia and Singapore in the past ten years^11,19,20,23,24^. Despite their relative susceptibility to antibiotics, the success of the ST22-SCC*mec* IV clone in displacing the more resistant ST239-SCC*mec* III clone warrants vigilance, particularly with the recent rise of hypervirulent ST22-SCC*mec* IV subclones that harbor the *pvl* and *tst* toxin genes in China and Japan^53,54^. The emergence of the Indian-origin clones ST772, and particularly ST672 in 2020 highlights their potential to spread and affirms the need for continuous monitoring by WGS.

## Methods

### Collection and selection of isolates

From July 2016 until December 2020, a total of 197 MRSA isolates were collected from various clinical specimens from the Microbiology Laboratory of Hospital Sultanah Nur Zahirah (HSNZ), which is the main referral and tertiary hospital of Terengganu, a state located in the eastern coast of Peninsular Malaysia. The collection of bacterial isolates was carried out with ethical approval from the Medical Research and Ethics Committee (MREC) of the Ministry of Health Malaysia (National Medical Research Registry nos. NMRR-15-2369-28,130 [IIR] and NMRR-19-3702-52104[IIR]). All methods were performed in accordance with the relevant guidelines and regulations.

All MRSA isolates were validated by the presence of the thermonuclease (*nuc*) gene^58^ and methicillin resistance (*mecA*) gene^59^ through PCR-amplification. Phenotypic resistance profiles against 24 antibiotics from 16 antibiotic classes using disk diffusion method and E-test strips, as well as double disk diffusion test (D-test) for macrolide-lincosamide-streptogramin B (MLS_B_) resistance phenotypes, i.e., inducible (iMLS_B_), constitutive (cMLS_B_), and macrolide-streptogramin (MS), were characterized as previously described^60^. From this collection, 88 MDR isolates, which were categorized as resistant to at least one agent in three or more antibiotic categories^61^ were selected for whole genome sequencing.

### DNA extraction and whole genome sequencing

Genomic DNA was extracted using Presto™ Mini gDNA Bacteria Kit (Geneaid Biotech Ltd., New Taipei City, Taiwan) according to the manufacturer’s instruction with lysostaphin (300 µg/mL) as an additional cell lysis reagent^13^. Construction of DNA libraries and whole genome sequencing using short-read, paired-end 150 bp (PE150) sequencing strategy were performed by commercial WGS service providers. Thirty-one isolates were sequenced on the Illumina platform using Illumina HiSeq 2500 (average sequencing coverage 250 ×) (Novogene, Singapore), and the remaining 49 isolates were sequenced on the DNBSEQ platform with BGISEQ-500 (average sequencing coverage 200 ×) (BGI, Shenzhen, China). An additional eight samples were sequenced using the Illumina MiSeq (average sequencing coverage 64 ×) under a collaborative program with the Kyushu Institute of Technology, Japan. Sequences were subjected to de novo assembly using Unicycler v0.4.8 (Wick et al., 2017). The quality of the assembled genomes was evaluated using QUAST v5.0.2^62^, where assemblies with > 200 contigs and N50 < 40,000 bp were excluded^63^. The assembled genomes were annotated using Prokka v1.13.3^64^.

### Identification of epidemiological markers, antimicrobial resistome and virulome

Molecular characterization of epidemiological marker elements such as staphylococcal cassette chromosome *mec* (SCC*mec*) types, multilocus sequence typing (MLST) sequence types and staphylococcal protein A (*spa*) types were determined from the assembled genomes using web-based databases, i.e., SCC*mec*Finder 1.2^65^, MLST 2.0^66^ and spaTyper 1.0^67^, respectively, which are available at the Center for Genomic Epidemiology (CGE) (http://www.genomicepidemiology.org/services/). Presence of acquired antimicrobial resistance genes and chromosomal mutations was identified using Resistance Gene Identifier (RGI) at the Comprehensive Antibiotic Resistance Database (CARD) (Alcock et al., 2020)] and ResFinder 4.1^68,69^, while VirulenceFinder 2.0^70^ was used to identify virulence genes.

### Determination of concordance rate between antimicrobial phenotypes and genotypes

The phenotypic resistance of the MRSA isolates was compared with the genomic prediction of antibiotic resistance determined from their respective assembled genomes. The concordance rate was calculated as the percentage of concordant isolates from the total number of isolates, with concordant isolates defined as those having phenotypic resistance and the corresponding resistance determinants in their genomes^41^.

### Pan genome and phylogenetic tree analysis

The annotated general feature format (GFF) files derived from Prokka were used as input for the pan genome pipeline using Roary^71^ with parameters set at 95% amino acid sequence identity and presence in 99% isolates to define a core gene. A maximum likelihood phylogenetic tree was constructed from the derived core genome alignment using FastTree 2.1.10^72^, with 100 bootstraps under the generalized time reversible (GTR) model. The phylogenetic tree also included the genome sequences of 18 Malaysian MRSA isolates available in the NCBI GenBank database. Among these isolates, 15 were clinical MRSA isolates obtained from various specimens, while three isolates were obtained from the nasal swabs of healthy student volunteers. The collection of these isolates spanned from 2009 to 2017. Further information about these isolates is included in Supplementary Table S1. The resulting phylogenetic tree was visualized and edited using iTOL v6 (https://itol.embl.de/)^73^. The accession numbers for all 88 MRSA genomes that were sequenced in this study are available in GenBank under BioProject accession number PRJNA722830.

### Supplementary Information

Supplementary Information.

## Data Availability

The genomic data of this study were submitted and deposited in NCBI GenBank database under BioProject No. PRJNA722830. Details regarding the 18 Malaysian database isolates are provided in Supplementary Table S1 while details regarding assembly qualities and statistics are provided in Supplementary Table S2.
